# Emergence of plasmid-mediated fosfomycin resistance among *Escherichia coli* harboring *fosA4*, *tet*(X4), and *mcr-1* genes in wild birds

**DOI:** 10.1128/msystems.01673-24

**Published:** 2025-03-13

**Authors:** Asim Munir, Xiaoyu Lu, Farwa Humak, Cemil Kürekci, Muhammad Shahid Mahmood, Sehrish Gul, Zhiqiang Wang, Mashkoor Mohsin, Ruichao Li

**Affiliations:** 1 Jiangsu Co-innovation Center for Prevention and Control of Important Animal Infectious Diseases and Zoonoses, College of Veterinary Medicine, Yangzhou University, Yangzhou, China; 2 College of Pharmacy and Chemistry and Chemical Engineering, Taizhou Univeristy, Taizhou, Jiangsu, China; 3 Institute of Microbiology, University of Agriculture, Faisalabad, Pakistan; 4 Department of Veterinary Medicine and Animal Productions, University of Naples Federico II 9307 , Naples, Italy; 5 Department of Food Hygiene and Technology, Faculty of Veterinary Medicine, Hatay Mustafa Kemal University, Hatay, Turkey; Pacific Northwest National Laboratory, Richland, Washington, USA

**Keywords:** antimicrobial resistance, wild birds, *E. coli*, *fosA4*, *mcr-1*, *tet*(X4)

## Abstract

Fosfomycin represents a last-line reserve antibiotic for the treatment of infections caused by multidrug-resistant (MDR) bacteria. Nevertheless, the advent of plasmid-mediated fosfomycin resistance among bacteria from humans and food animals incurs great concern. This study reports the detection and genomic portrait of the plasmid-mediated fosfomycin resistance gene, *fosA4*, amid *Escherichia coli* from wild birds co-harboring plasmid-mediated tigecycline resistance gene*, tet*(X4), and colistin resistance gene, *mcr-1*. A total of 100 samples from fecal droppings of wild birds in the urban parks in Faisalabad, Pakistan were subjected for the isolation and characterization of fosfomycin-resistant *E. coli*. The *fosA4* gene was identified in 11 (11%) of the *E. coli* isolates, and all exhibited an MDR phenotype. Genome sequencing confirmed that all the *fosA4*-positive isolates also co-harbored the mobile tigecycline resistance *tet*(X4) gene on a large MDR IncFII plasmid. One isolate PKF8 belonging to ST48 also co-carried the colistin resistance gene *mcr-1* on the IncHI2 plasmid. To the extent of our knowledge, this is the first discovery of *E. coli* isolates in wild birds co-harboring the *mcr-1*, *fosA4*, and *tet*(X4) genes. The emergence of these pivotal antimicrobial resistance genes in wild birds native to South Asia with their close association to humans and animals is alarming. Our findings highlight the urgent need for further surveillance of bacterial resistance to last-resort antibiotics in the clinics, animal farming, and environment with the One Health approach.

**IMPORTANCE:**

The global spread of the plasmid-mediated fosfomycin resistance gene *fosA4* bearing *Escherichia coli* strains incurs a public health concern. However, research focusing on the pervasiveness of *fosA4*-positive isolates in wild birds is still rare, and to the best of our knowledge, this is the first documentation from South Asia highlighting the concurrent presence of the *fosA4*, *mcr-1*, and *tet*(X4) genes within *E. coli* isolates recovered from fecal samples of wild birds in Pakistan. This co-existence of ARGs along with phylogenetic analysis revealed that MDR plasmids carried by *E. coli* isolates have the ability to spread horizontally between wild birds, food animals, and humans. Co-existence of *fosA4*, *tet*(X4), and *mcr-1*-carrying plasmids is worrying and warrants further investigation.

## INTRODUCTION

Antimicrobial resistance (AMR) embodies a significant public health concern that impedes the ability to prevent and treat bacterial infections (1, 2). The global rise in multidrug-resistant (MDR) bacteria is accompanied by an increase in the utilization of antimicrobials in food animals for therapeutic or prophylactic purposes. In some food industries, antibiotics are routinely used to promote animal growth and immune function at sub-therapeutic levels (3). Abundant use and misuse of antibiotics are not restricted to food production: irrational prescriptions in human medicine also contribute to the growing status and number of MDR pathogens (4). The manifestation of clinically relevant MDR bacteria in the environment is a developing disquiet, and wild birds have been considered as a sentinel for AMR surveillance (5). The presence of AMR bacteria in wild birds and their droppings poses a potential public health risk. They may be considered reservoirs of AMR and a source for the spread of resistant bacteria or antimicrobial resistance genes (ARGs) to bacteria that can colonize or infect humans and other animals (6, 7). A One Health approach, which integrates surveillance and research across animal, human, and environmental niches, is required to assess and address the significant global increase in AMR (8, 9). The prevalence of genes conferring resistance to various last-resort antibiotics, including colistin, carbapenems, and tigecycline, is increasing. As a result, the availability of effective antimicrobials is decreasing. Consequently, the efficacy of older antibiotics, such as fosfomycin, is being re-evaluated and regaining clinical interest against some MDR pathogens (10). Fosfomycin is the drug of choice for the treatment of urinary tract infections (UTIs) in humans and has recently been listed by the World Health Organization as the highest-priority critically important antimicrobials (11). Nevertheless, resistance to fosfomycin has also been documented in *E. coli* sourced from human, dairy cattle, and migratory wild birds (12–14). In particular, the advent of plasmid-mediated fosfomycin-resistant genes among gram-negative bacteria represents a significant challenge. There are three primary mechanisms that *E. coli* uses to resist fosfomycin. The first is via the mutation of transporter genes *glpT* and *uhpT*, thereby reducing the ability of fosfomycin to accumulate in cells. The second is a regulator mechanism in which the overexpression of the fosfomycin target gene *murA* promotes survival. The final mechanism is the acquisition of plasmid-mediated glyoxalase and metalloenzymes that accelerate the conjugation of glutathione to the epoxide ring of the antibiotic and hinder its activity (FosA1-10, FosC2, and FosL1-2) (15, 16). The plasmid-mediated *fosA3* gene and the less frequently observed variant *fosA4* have been reported in different sources, including human, animal, and environmental *E. coli* isolates (17–19). These plasmid-mediated genes provide a high level of resistance in *Enterobacterales* and are believed to originate from *Kluyverra georgiana* (20, 21). The natural co-existence of humans and birds has been disrupted by the advent of industrialization, globalization, climate change, agriculture, urbanization, and habitat destruction (22). The ability of birds to travel long distances in short periods makes them potential vectors for the transmission of resistant bacteria and ARGs (23). Indeed, the aforementioned disruptions have led to more complex interactions with humans, and the prevalence of newly recovered zoonotic pathogens from birds has increased over time (24). Furthermore, climate change has led to an animal and water bird reliance on artificial habitats by water birds, including wastewater treatment works and landfills. These habitats are contaminated with MDR bacteria that can colonize wild birds (25, 26). It has also been demonstrated that birds can acquire antibiotic-resistant bacteria during their migration from human-influenced environments or through horizontal gene transfer from contact with other birds in similar environments (27, 28). Thus, the evolution of plasmid-mediated fosfomycin resistance genes, particularly *fosA3* and *fosA4*, in wild birds could represent a meaningful reservoir for the dissemination of resistance to highest priority critically important antimicrobials.

However, data on the existence of fosfomycin resistance in *E. coli* from wild birds are still very limited. Hence, the purpose of this study is to probe and characterize the fosfomycin-resistant *E. coli* isolated from urban wild birds in the city of Faisalabad, Pakistan.

## MATERIALS AND METHODS

### Sample collection and strain isolation

In this cross-sectional study, a total of 100 fresh fecal droppings of wild birds, including kites and house crows foraging in public parks with known anthropogenic interaction, were randomly collected from different locations in Faisalabad, Pakistan during May 2022 (29). A single, fresh, isolated fecal drop was thought to be representative of individual birds and collected using a sterile charcoal cotton swab. The collected samples were then mixed with nuclease-free water or PBS buffer, and 50–100 µL was inoculated into 1.5 mL of brain heart infusion broth supplemented with fosfomycin. The inoculated samples were then incubated with shaking for 6 h at 37°C. In order to isolate *E. coli*, all specimens were cultured on UTI ChromoSelect agar (Merck, Darmstadt, Germany) enriched with 32 µg/mL of fosfomycin along with glucose 6-phosphate and subsequently incubated at 37°C for 18 h. This incubation period was employed to facilitate the isolation of fosfomycin-resistant *E. coli* strains. The bacterial species was confirmed using matrix-assisted laser desorption/ionization time-of-flight technology (Bruker). PCR was employed to screen for the presence of the *fosA3* and *fosA4* genes in fosfomycin-resistant isolates using primers that had been formerly reported (30).

### Antimicrobial susceptibility testing

The minimum inhibitory concentrations (MICs) of 14 antimicrobial agents, namely, tetracycline (TET), ampicillin (AMP), meropenem (MEM), tigecycline (TIG), kanamycin (KAN), florfenicol (FFC), gentamicin (GEN), colistin (CST), streptomycin (STR), ceftriaxone (CTX), doxycycline (DOX), amoxicillin (AMC), enrofloxacin (ENR), and rifampicin (RIF), were obtained from all *fosA*-positive isolates in two replicates. The results were calculated in accordance with the Clinical and Laboratory Standards Institute guidelines (31). For colistin and tigecycline resistance MIC, interpretation was based on breakpoint values established by the European Committee on Antimicrobial Susceptibility Testing (EUCAST) (32). *E. coli* ATCC 25922 was used as a quality control strain.

### Whole-genome sequencing and bioinformatics analysis

The FastPure Bacterial DNA Isolation Mini Kit (Vazyme, China) was employed to extract genomic DNA from 11 fosfomycin-resistant *E. coli* isolates in a highly efficient manner. Following centrifugation of 2 mL bacterial cultures at 13,500 *g* for 1 min, the bacterial pellet was lysed with proteinase K and treated with RNase to prevent any contamination with RNA. This column-based method ensured the extraction of high-quality genomic DNA, which was confirmed by the Colibri LB 915 spectrophotometer (Titertek-Berthold, Germany). Furthermore, gel electrophoresis was employed to evaluate the concentration and quality of the extracted DNA.

The extracted DNA was subjected to a paired-end sequencing technique using the (2 × 150 bp) Illumina HiSeq2500 platform (Illumina, San Diego, CA, United States). SPAdes v3.13.1 was employed to generate draft genome assemblies from short Illumina raw reads, with predefined parameters (33). The draft genome assemblies were used to determine the multi-locus sequence types (MLST) using an online tool (https://github.com/tseemann/mlst). The acquired ARGs, plasmid replicon types, and insertion sequences were determined using ResFinder v4.1, PlasmidFinder v2.1, and Isfinder v2.1, respectively (34–36). The draft assembled contigs underwent annotation for subsequent analysis utilizing Prokka v1.12 (37). Following annotation, phylogenetic analysis of the annotated contigs was conducted based on single nucleotide polymorphism (SNP) distance of the genome using Roary and FastTree (38, 39). The constructed phylogenetic tree was visualized using the online software, iTOL v5 (https://itol.embl.de/itol.cgi) (40). A pairwise distance matrix of SNPs based on the core genome was created using snp-dists v0.7.0 (https://github.com/tseemann/snp-dists). In accordance with the results of the phylogenetic analysis, the genomic DNA from three strains was subjected to long-read sequencing using the Oxford Nanopore Technologies MinION platform (41). The complete genomes of three strains were assembled by integrating short Illumina reads with long Nanopore reads using the *de novo* assembler Unicycler v0.4.8 (42). Followed by hybrid assembly, the complete circular genomes of the selected strains were annotated via the rapid annotation using subsystems technology (https://rast.nmpdr.org/rast.cgi) (43). In order to reveal the genetic context, circular and linear comparisons were generated between fosfomycin-resistant strains from this study and online available sequences in the National Center for Biotechnology Information (NCBI) database using BRIG v0.95 (44) and Easyfig v2.2.3 (45), respectively.

## RESULTS

### Identification of *fosA4*-positive *E. coli* isolates

**Fig 1:**
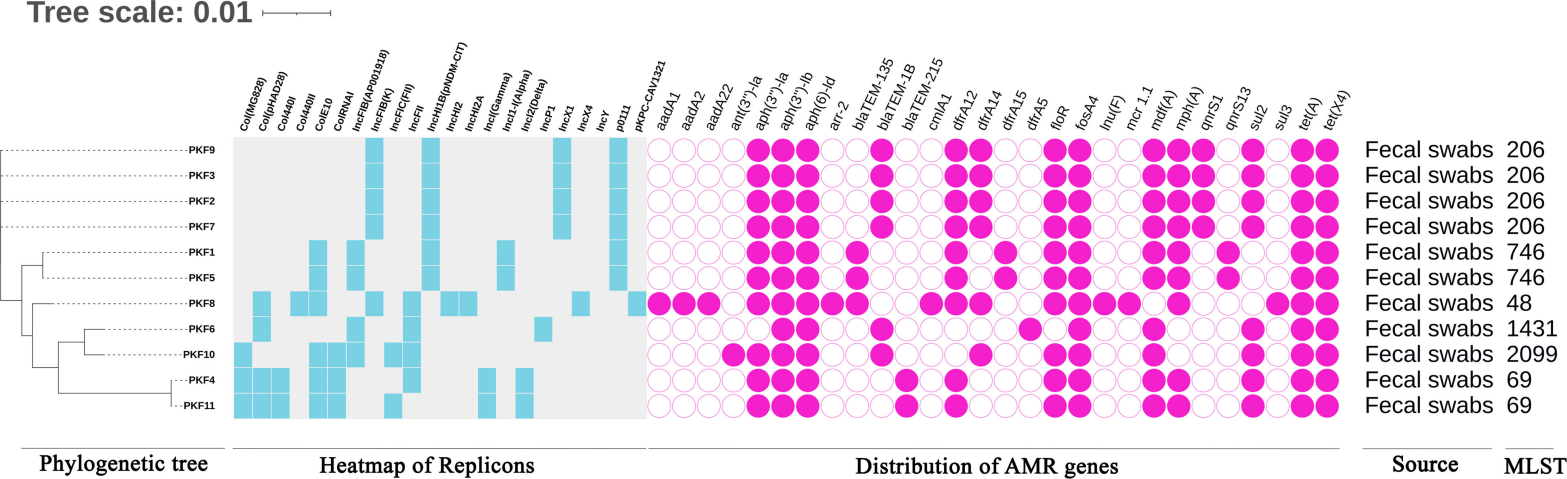
The figure presents a phylogenetic tree constructed from genomic data of 11 *fosA4*-positive *E. coli* isolates derived from fecal samples of wild birds. Additionally, the distribution of resistance genes, replicons, and sequence types is depicted. Replicon presence is denoted by light blue coloring, while absence is indicated by light gray. The presence of antimicrobial resistance (AMR) genes is marked by pink circles.

A total of 11 *fosA4*-positive *E. coli* isolates were identified from fecal droppings of wild birds using PCR. No *fosA3* positive isolates were detected. Interestingly, all *fosA4*-positive isolates also co-harbored the tigecycline-resistant gene *tet*(X4). In addition, one isolate also carried the colistin-resistant gene *mcr-1* in addition to *fosA4* and *tet*(X4) (Fig. 1).

### Antimicrobial resistance profile of *fosA4*-positive isolates

Antimicrobial susceptibility profiling depicts that all *fossA4*-positive isolates (100%) were also resistant to tigecycline (2–8 µg/mL), while four isolates were resistant to colistin, with MICs ranging from 4 to 8 µg/mL. In addition, all isolates were highly susceptible to meropenem while showing moderate to high resistance to TET, AMP, KAN, FFC, GEN, STR, CTX, DOX, AMC, ENR, and RIF (Table 1).

**TABLE 1:** MICs (µg/mL) of 11 *fos*A4-positive *E. coli* isolates against 14 antimicrobials^*a*^

Strain ID	MIC (µg/mL) of:													
	TET	AMP	MEM	TIG	KAN	FFC	GEN	CST	STR	CTX	DOX	AMC	ENR	RIF
PKF1	>128	>128	≤0.25	8	128	128	2	≤0.25	128	1	64	128	4	16
PKF2	>128	>128	≤0.25	0.5	128	128	4	≤0.25	128	1	64	128	8	8
PKF3	64	>128	≤0.25	8	128	128	2	≤0.25	128	1	128	128	8	16
PKF4	64	>128	≤0.25	2	128	128	8	8	128	≤0.25	32	128	16	8
PKF5	64	>128	≤0.25	8	128	128	4	0.5	128	1	128	128	16	32
PKF6	>128	>128	≤0.25	8	8	8	4	≤0.25	128	0.5	64	128	128	8
PKF7	>128	>128	≤0.25	4	128	128	4	≤0.25	128	1	64	128	16	16
PKF8	>128	>128	≤0.25	8	128	128	4	4	128	0.5	64	128	64	128
PKF9	>128	>128	≤0.25	2	128	128	8	4	128	2	64	128	8	16
PKF10	>128	>128	≤0.25	8	128	128	2	≤0.25	128	0.5	64	128	32	16
PKF11	>128	>128	≤0.25	4	128	128	2	4	128	≤0.25	32	128	16	32
ATCC 25922	1	8	≤0.25	≤0.25	8	4	4	≤0.25	4	≤0.25	2	2	≤0.25	4

^*a*^ TET, tetracycline; AMP, ampicillin; MEM, meropenem; TIG, tigecycline; KAN, kanamycin; FFC, florfenicol; GEN, gentamicin; CST, colistin; STR, streptomycin; CTX, ceftriaxone; DOX, doxycycline; AMC, amoxicillin; ENR, enrofloxacin; RIF, rifampicin.

### Phylogenetic analysis and clonal relationship

**Fig 2:**
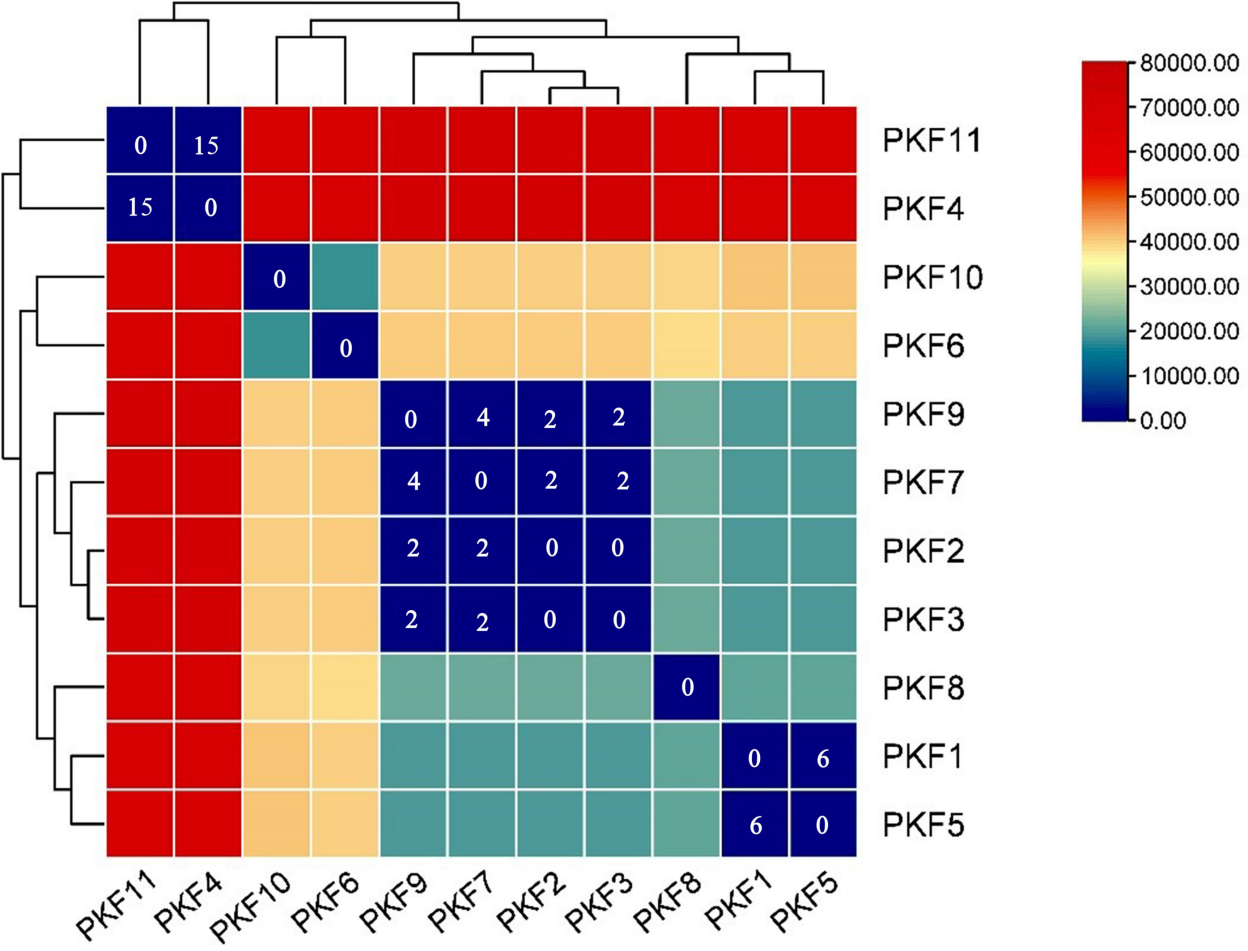
Pairwise distance matrix of 11 *fosA4*-positive isolates based on single-nucleotide polymorphisms. The spectrum of color transition ranging from blue to orange indicates the incremental variation in SNP counts, spanning from 0 to 80,000 across the strains.

Whole-genome sequencing (WGS) analysis provided in-depth information of 11 *fosA4* positive isolates and revealed their phylogenetic lineage and clonal relationship. The phylogenetic tree exhibited that all *fosA4* carrying *E. coli* were distributed among six different ST types: ST206 (*n* = 4), ST746 (*n* = 2), ST69 (*n* = 2), and one each of ST48, ST1431, and ST2099, supporting the prevalence of limited clonal and non-clonal spreads of the *fosA4* gene in wild birds simultaneously (Fig. 1). PKF2, PKF3, PKF7, and PKF9 belonged to ST206 and exhibited SNP distances ranging from 0 to 4, suggesting a close relationship indicative of a recent common ancestry or minimal evolutionary divergence. Conversely, isolates PKF1 and PKF5, which belong to ST746, have a SNP distance of only 6. In addition, the SNP distance between isolates PKF4 and PKF11, which both belong to ST69, is 15. The phylogenetic relationship and diversity of the MLST types suggest that the *fosA4* and *tet*(X4) harboring isolates in wild birds from Pakistan were assorted, but clonal dissemination also existed (Fig. 2).

### Prevalence of acquired antibiotic resistance genes

The prevalence of ARGs in 11 isolates exhibits great diversity. Multiple ARGs were detected, and all the isolates were declared as MDR pathogens. In addition to *fosA4*, 28 other resistance genes were detected in 11 *E. coli* isolates, conferring resistance to various antibiotics, such as aminoglycosides, β-lactams, phenicol, macrolides, fluoroquinolones, tetracycline, tigecycline, polymyxins, lincosamides, sulfonamides, and trimethoprim. Among all the isolates, PKF8 harbored the highest number of resistance genes, that is, 19/28 (67.85%). The most predominant ARGS were *tet*(A), *tet*(X4), *aph(3")-Ib*, and *aph(6)-Id* conferring resistance to tetracycline, tigecycline, and aminoglycosides (*n* = 11, 100%), respectively. Apart from *aph(3")-Ib* and *aph(6)-Id,* five other genes were identified that also confer resistance to aminoglycosides: *aadA1* (*n* = 1), *aadA2* (*n* = 1), *aadA22* (*n* = 1), *ant(3')-Ia* (*n* = 1), and *aph(3')-Ia* (*n* = 10). All isolates, except PKF6, carried at least one phenicol resistance gene. Interestingly, only one isolate, PKF8, harbored the colistin resistance *mcr-1* gene. Besides the five trimethoprim resistance genes *dfrA5* (*n* = 1), *dfrA12* (*n* = 9), *dfrA14* (*n* = 6), *dfrA15* (*n* = 2), and three sulfonamide resistance genes *sul1* (*n* = 3), two quinolone and two macrolide-resistant genes *qnrS1* (*n* = 4), *qnrS13* (*n* = 2), *mdf*A (*n* = 10), and *mph*A (*n* = 9) were also detected. Multiple variants of phenicol and sulfonamide resistance genes, including *cmlA1* (*n* = 1), *flor* (*n* = 10), *sul2* (*n* = 8), and *sul3* (*n* = 1), were identified, while rifampicin-resistant *arr-2* (*n* = 1) and lincosamide-resistant gene *lnu*(f) (*n* = 1) were also identified. However, all isolates harbor at least one variant of β-lactam resistance genes, including *bla*_TEM-1B_ (*n* = 6), *bla*_TEM-135_ (*n* = 3), and *bla*_TEM-215_ (*n* = 2); therefore, all isolates exhibit a high degree of resistance and MICs against amoxicillin and ampicillin (Table 1). There was huge diversification in replicon types, and 22 different types of replicons were found in 11 isolates. Each strain contained at least two replicon sequence types. IncHI1B (pNDM-CIT), ColE10, and p0111 were the most prevalent plasmid incompatibility groups found in six isolates (Fig. 1).

### Genomic characterization of *fosA4* and *mcr-1* isolates

**Fig 3:**
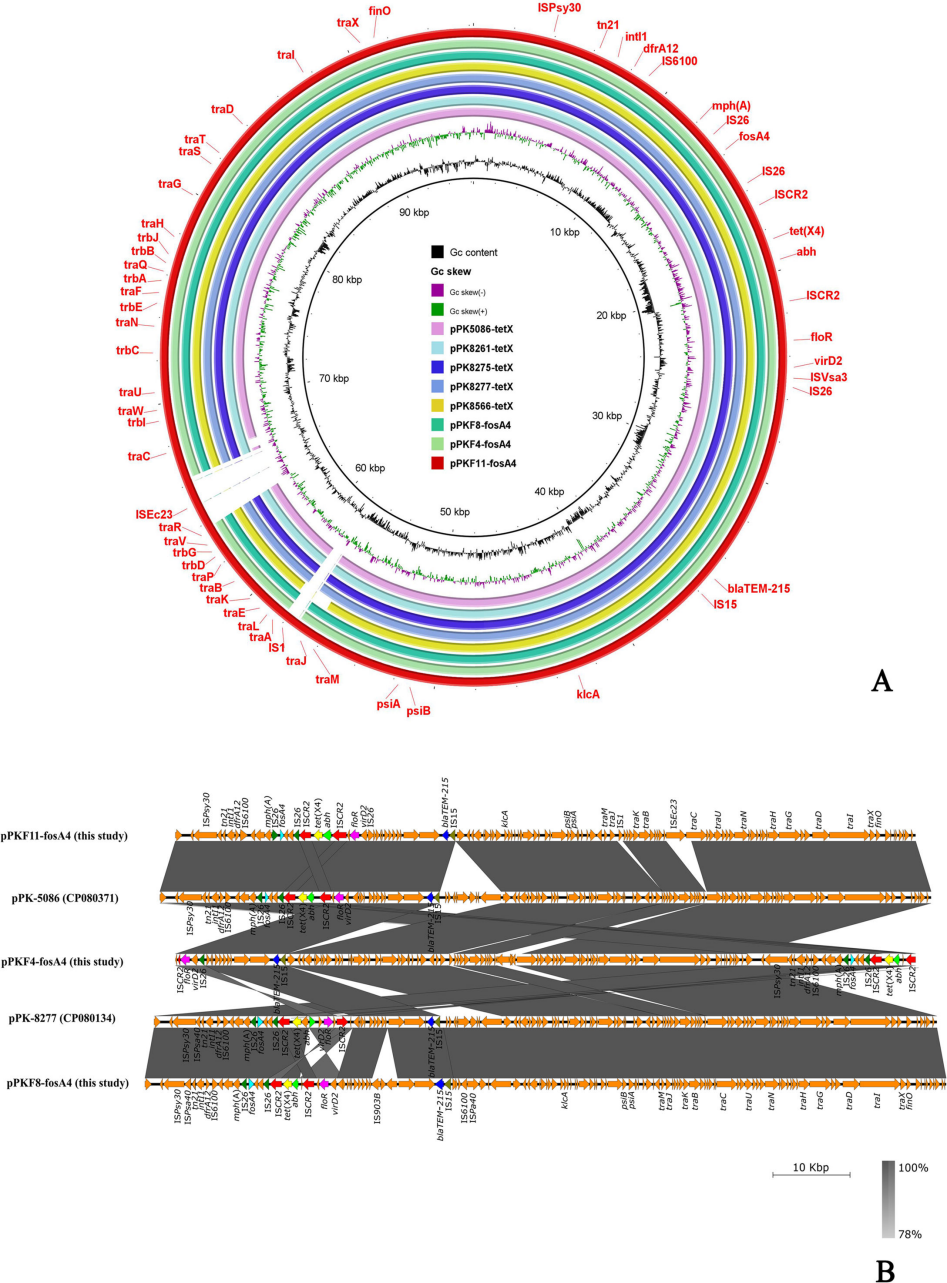
(**A**) Circular comparison of eight IncFII *fosA4* was performed using BRIG v0.95. Three IncFII *fosA4*-harboring plasmids from this study were comparatively analyzed with five plasmids, namely, pPk5086, pPK8566, pPK8277, pPk8261, and pPK8275-*fosA4*, in the NCBI NR database. (**B**) Linear comparison drawn using Easyfig v2.2.3 between three *fosA4* plasmids from this study with two reference plasmids pPK-5086 and pPK-8277 sourced from the NCBI database.

**Fig 4:**
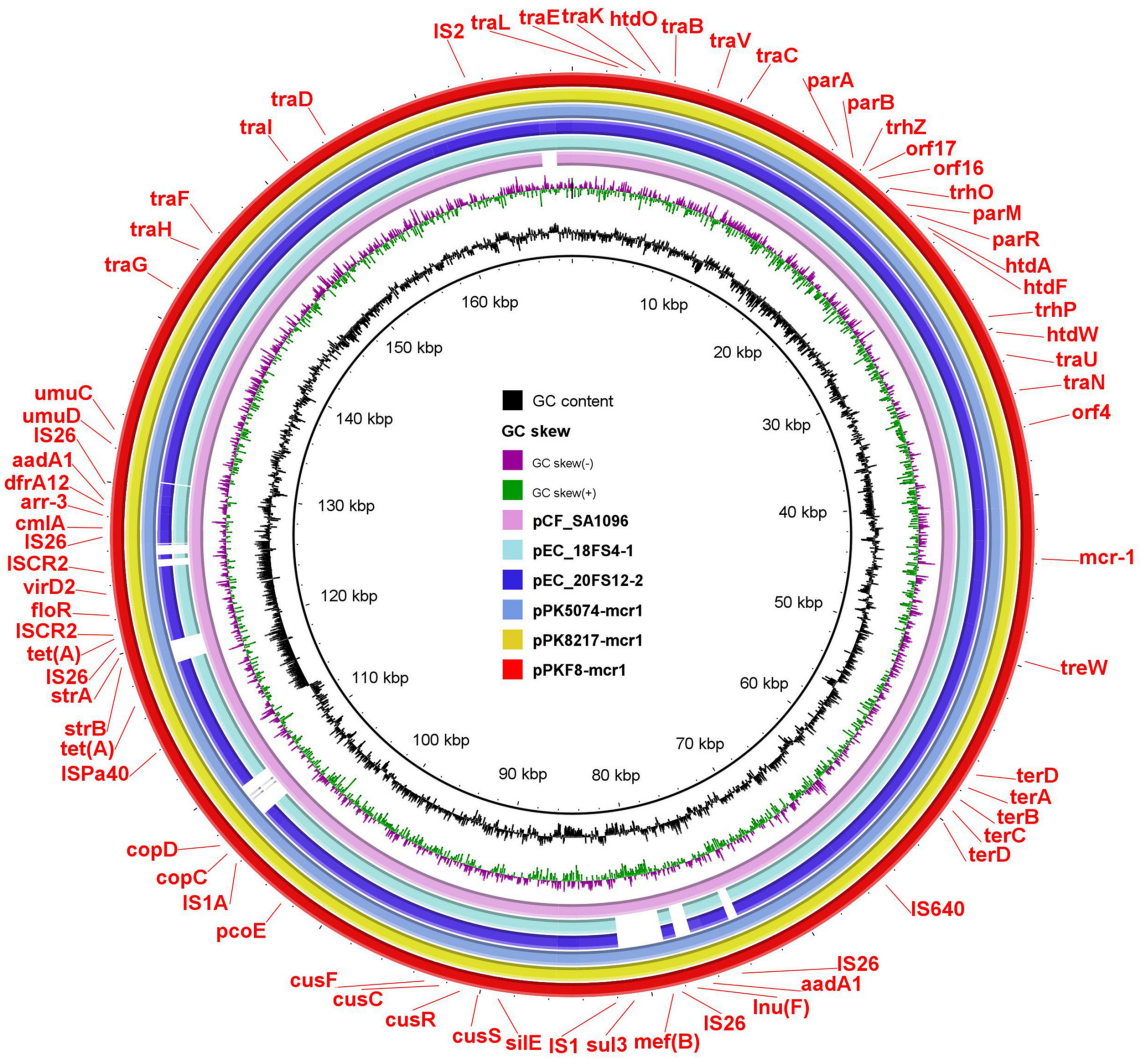
Circular comparison of six IncHI2 plasmids harboring the *mcr-1* gene was performed using BRIG software v0.95. The IncHI2 plasmid designated as pPKF8-mcr in this investigation was comparatively analyzed with five other plasmids, namely, pCFSA1096, pEC_18FS4-1, pEC_20FS12-2, pPK8217, and pPK5074-MCR1, sourced from the NCBI NR database.

In accordance with phylogenetic analysis, the genomic DNA of three isolates (PKF4, PKF8, and PKF11) was long-read-sequenced using the Oxford Nanopore MinION platform, and preliminary details regarding the whole genome were summarized in Table 2. All these MDR isolates also confer chromosomally encoded resistance against multiple antibiotics. In PKF4, PKF8, and PKF11, the *fosA4*genes were present on the MDR IncFII plasmid, which also co-carried several ARGs, such as *dfrA12*, *mph* (A), *tet*(X4), *floR,*, and *bla*_TEM-215_. Genomic analysis of three *fosA4*-bearing plasmids exhibits a high degree of genetic similarity in contrast with five reported plasmids from the NCBI database: pPk5086-tetX (CP080371) with 99.99% identity at 95% coverage, pPK8566-tetX (CP080175) with 100% identity at 95% coverage, pPK8277-tetX (CP080134) with 99.97% identity at 96% coverage, pPk8261-tetX (CP080156) with 99.97% identity at 96% coverage, and pPK8275-tetX (CP080164) with 99.97% identity at 96% coverage from multiple sources, including clinical, chicken meat, chicken liver, and chicken cloacal swab samples, respectively (Fig. 3A). Figure 3B shows that three strains from this study and two plasmids, pPK5086-tetX (CP080371) and pPK8277-tetX (CP080134), shared the same genetic makeup as the *IS26* present upstream and downstream of the *fos*A4 gene. Along with that, the *ISCR2* was present upstream and downstream of *tet*(X4) *ΔISCR2-ΔvirD2-abh-tet*(X4) *ΔISCR2* that depicts that these insertion sequences act as a mobilizer and help to mobilize these genes within the plasmids. In addition to the *fosA4* one strain, PKF8 also co-carried the colistin-resistant *mcr-*1 gene on the IncHI2 (~252 Kb) plasmid. Blastn analysis showed that pPKF8-*mcr-1* shared a high genetic resemblance with 99% identity at 100% coverage to pCSFA1096 (CP033347) from *Salmonella* while showing 100% identity at 100% coverage to pPK8217 (CP080121) and pPK5074 (CP072803) from *E. coli* isolates recovered from chicken and clinical isolates, respectively (Fig. 4).

**TABLE 2:** Genomic characterization of *E. coli* PKF4, PKF8, and PKF11 finished by hybrid assembly approach^*a*^

Strain ID	MLST	Component	Replicon type	Size (bp)	Resistance genes
PKF4	ST 69	PKF4-chromosome	-	5,362,652	*aph(3'')-Ib*, *aph(3')-Ia, aph(6)-Id, floR*, *sul2, tet*(A), *bla*_Ec-8_
		pPKF4-145kb	IncI(Gamma)	145,190	None
		pPKF4-fosA4	IncFII	96,210	*tet*(X4), *fosA4*, *mph*(A), *dfrA12*, *floR*, *bla*_TEM-215_
		pPKF4-8kb	ColRNAI	8,945	None
		pPKF4-9kb	Col(pHAD28)	9,345	None
PKF8	ST 48	PKF8-chromosome	-	4,584,547	*bla*_Ec-15_
		pPKF8-*mcr-1*	IncHI2	252,734	*mcr-1*, *aadA22*, *lnu(F)*, *sul3*, *tet*(A), *aph(6)-Id*, *floR*, *arr-2*, *dfrA14*
		pPKF8-120kb	IncFIB(K)	126,076	*tet*(A), *bla*_TEM-135_, *dfrA15*, *aph(3')-Ia*, *sul3*, *aadA1*, *cmlA1*, *aadA2*, *aph(3'')-Ib*, *aph(6)-Id*
		pPKF8-fosA4	IncFII	104,161	*tet*(X4), *fosA4*, *mph*(A), *dfrA12*, *floR*, *bla*_TEM-135_
		pPKF8-91kb	IncFII	91,466	None
		pPKF8-41kb	IncX4	41,455	None
		pPKF8-4kb	Col(pHAD28)	4,461	None
PKF11	ST 69	PKF11-chromosome	-	5,363,334	*bla*_EC-8_, *aph(3'')-Ib*, *aph(6)-Id*,* tet*(A), *floR*, *aph(3')-Ia*, *aph(6)-Id*, *aph(3'')-Ib*, *sul2*
		pPKF11-144kb	IncI(Gamma)	144,862	None
		pPKF11-fosA4	IncFII	96,209	*dfrA12*, *mph*(A), *fosA4*, *tet*(X4), *floR*, *bla*_TEM-215_
		pPKF11-89 kb	ColRNAI	8,910	None
		pPKF11-41kb	Col(pHAD28)	4,677	None
		pPKF11-4kb	ColRNAI	4,593	None
		pPKF11-1kb	Col(MG828)_	1,549	None

^*a*^ -, not applicable.

## DISCUSSION

This study resulted in the first report of the co-existence of *mcr-1*, *tet*(X4), and *fosA4* in *E. coli* isolates recovered from wild birds in Pakistan. In our study, fosfomycin-resistant *E. coli* isolates exhibited resistance against most of the tested antibiotics, including tigecycline and colistin. Previous studies also showed MDR patterns among fosfomycin-resistant *Enterobacterales* from environmental samples in Switzerland (46). To date, a number of *fosA* variants have been reported in *Enterobacterales*. This is in accordance with a study conducted in Spain that reported that 3 of 55 (5.5%) *E. coli* isolates recovered from hospitalized patients harbored fosfomycin-resistant gene variants *fosA3*, *fosA4*, and *fosA6* (18). A similar study conducted in Portugal reported a much lower clinical incident of *fosA4* (0.02%) in a collection of 19,186 *E. coli* (47). Here, fosfomycin resistance was mainly attributed to *fosA4,* whereas previous studies reported *fosA3* as the most prevalent variant in *E. coli* strains that were resistant to fosfomycin (48). Two studies from China reported the prevalence of the *fosA3* gene among CTX-M and carbapenemase-producing *E. coli* as 27.4% and 10.3%, respectively (49, 50). Our approach to prioritize the study of fosfomycin resistance carried by wild birds resulted in the recoveries of MDR isolates, which speaks generally to the co-occurrence of multiple ARGs encoded by plasmids. The results also support the notion that the isolation of fosfomycin-resistant *E. coli* from readily accessible environmental samples is a relevant sentinel of clinical AMR. The co-existence of fosfomycin-resistance genes with other ARGs builds on our recent work in the area. We reported a *tet*(X4)-positive *E. coli* obtained from a wild bird in Pakistan (29). The co-existence of *fosA4* and *tet*(X4) in *E. coli* isolates was distributed across six different STs, the most important being ST48 and ST69, which have also previously been reported from various sources, including wastewater treatment plant (WWTP), chicken, human, and wild birds associated with fosfomycin-resistant *E. coli* (12, 13, 15, 51). We found that 11% (11/100) of *E. coli* were *fosA4* and *tet*(X4) positive, and worryingly, one isolate also co-carried the *mcr-1* gene. The presence of *fosA4* harboring ST48 *E. coli* isolates in wastewater and the coexistence of *fosA4* and *mcr-1* in *E. coli* isolates recovered from chicken, pig, and even clinical samples that exhibit diversified patterns of STs: ST48, ST69, ST155, ST216, and ST 3944 reveal that these genes are horizontally disseminated in the environment and require critical preventive measures to check the spread of *fosA4* and *mcr-1* co-harboring bacterial isolates (13, 15, 19, 52, 53). Our results from wild birds are in line with what has been reported in food animals, albeit the wild samples showing a lower overall frequency than those recovered from clinical cases. For example, another study from Pakistan reported the co-existence of *tet*(X4), *mcr-1*, and *fosA4* in 11% (4/36) *tet*(X4)-positive *E. coli* isolates recovered from chickens (13). The co-existence of tetracycline, colistin, and fosfomycin resistance was also observed in Egypt at 12.5% (4/50) in *E. coli* isolated from chickens, though *tet*(X7) rather than *tet*(X4) was present in these strains (52). The elevated incidence of *fosA4* observed in our study suggests the potential for regional variations in the incidence of plasmid-mediated fosfomycin resistance genes. On the contrary, the prevalence of *fosA4* is much higher in wastewater as compared to chicken *E. coli* isolates. According to a study conducted in Türkiye reporting on *E. coli* collected from WWTP and hospital sewage, 18/29 (62%) *fosA4* and 10/29 (34.4%) *fosA3* were positive, while another study in Türkiye documented *fosA4* and *fosA3* genes in 10/21 (47.6%) and 7/21 (33.3%) *E. coli* isolates from raw chicken meat, respectively (15, 54). The prevalence of the *fosA4* gene across human, animal, and environmental sectors underscores the potential for its dissemination within the One Health framework, possibly facilitated via the food chain and the environment. Wild and migratory birds are implicated as primary vectors, suggesting their significant role in potential AMR transmission pathways. In this study, *fosA4* was present on conjugative plasmid IncFII, along with *tet*(X4) and *bla*_TEM-215_, recapitulating previous reports from chickens (13). The results also show some geographical variation from previous work in China, where the occurrence of an IncFII plasmid harboring four resistance genes (*fosA3*, *bla*_CTX-M_, *bla*_TEM_, and *rmtB*) has been documented in *E. coli* isolates from chickens and ducks across the country (55). These plasmids also encoded for an α/β-hydrolase (labeled as *abh* in Fig. 3A and B throughout) that was recently implicated in macrolide resistance and named *estT* (56). The genetic neighborhood of the *fosA4* gene reveals that it exists within a region flanked up and downstream by *IS26*. The role of this insertion sequence in the mobilization of this gene has been reported previously (19, 57, 58). The linkage between *fosA4* and *IS26* transposases facilitates an expanded dissemination potential through translocation of resistance to other plasmid sequences or integration into the chromosome. Continuous surveillance and genomic analyses are imperative to evaluate the ramifications of fosfomycin resistance, whether plasmid or chromosome-associated, or whether on human, animal, or environmental health within the context of One Health.

### Conclusion

To the best of our knowledge, this is the first documentation from South Asia that highlights the concurrent presence of the *fosA4*, *mcr-1*, and *tet*(X4) genes within *E. coli* isolates recovered from fecal samples of wild birds in Pakistan. This co-existence of ARGs, along with phylogenetic analysis, revealed that MDR plasmids carried by *E. coli* isolates have the ability to spread horizontally between wild birds, food animals, and humans. WGS analysis revealed that homologous genomic profiles of *fosA4*, *tet*(X4), and *mcr-1* were usually dispersed in isolates from chickens and humans. These findings suggest that wild birds are possible reservoirs that transfer ARGs among bacteria, colonizing in poultry and humans without geographical limitations, thereby promoting the dissemination of ARGs across the globe. Continued surveillance is necessary with the ambition of observing the prevalence and transmission dynamics of emerging ARGs, such as *fosA4* and *mcr-1*, in wild birds.

## Data Availability

The data sets presented in this study have been deposited in NCBI GenBank under BioProject ID PRJNA1112118.

## SUPPLEMENTAL MATERIAL

Supplemental material msystems.01673-24-s0001.xlsx Mean and standard deviation of two MIC repeats.

ASM does not own the copyrights to Supplemental Material that may be linked to, or accessed through, an article. The authors have granted ASM a non-exclusive, world-wide license to publish the Supplemental Material files. Please contact the corresponding author directly for reuse.
